# Prognostic value of meta-signature miRNAs in renal cell carcinoma: an integrated miRNA expression profiling analysis

**DOI:** 10.1038/srep10272

**Published:** 2015-05-14

**Authors:** Kun Tang, Hua Xu

**Affiliations:** 1 Department of Urology, Tongji Hospital, Tongji Medical College, Huazhong University of Science and Technology, Wuhan, China; 2 Institute of Urology, Tongji Hospital, Tongji Medical College, Huazhong University of Science and Technology, Wuhan, China

## Abstract

To identify a robust panel of microRNA (miRNA) signatures that can distinguish renal cell carcinoma (RCC) from normal kidney using miRNA expression levels. We performed a comprehensive meta-analysis of 29 published studies that compared the miRNA expression profiles of RCC tissues and adjacent normal tissues (NT) to determine candidate miRNAs as prognostic biomarkers for RCC. Using vote-counting strategy and robust rank aggregation method, we identified a statistically significant miRNA meta-signature of two upregulated (miR-21, miR-210) and three downregulated (miR-141, miR-200c and miR-429) miRNAs. X-tile plot was used to generate the optimum cut-off point for the 15 different deregulated miRNAs and Kaplan-Meier method was used to calculate CSS. In a cohort of 45 patients, the high expression of miR-21 (HR: 5.46, 95%CI: 2.02-53.39) and miR-210 (HR: 6.85, 95%CI: 2.13-43.36), the low expression of miR-141 (HR: 0.16, 95%CI: 0.004-0.18), miR-200c (HR: 0.08, 95%CI: 0.01-0.43) and miR-429 (HR: 0.18, 95%CI: 0.02-0.50) were associated with poor cancer-specific survival (CSS) following RCC resection. We also constructed a five-miRNAs-based classifier as a reliable prognostic and predictive tool for CSS in patients with RCC, especially in clear cell RCC (ccRCC) (HR: 5.46, 95% CI: 1.51-19.66). This method might facilitate patient counselling and individualise management of RCC.

Renal cell carcinoma (RCC) accounts for approximately 3% of all adult malignancies^1^, and its incidence has increased over the past two decades. Renal cell carcinoma (RCC) is a potentially curable disease, especially if the tumor is limited to the kidneys and no systemic metastatic spread has occurred by the time of diagnosis. Surgical resection is still the only definitive treatment for RCC, but after the curative nephrectomy, 20–40% patients will develop recurrence. Apart from surgery, it is both chemotherapy and radiotherapy resistant. Despite the potential for successful surgical removal of the tumor-bearing organ in localized stages and the likelihood of treatment success, the complications and long-term morbidity and mortality of RCC are difficult to accurately predict. The 5-year survival of RCC is estimated to be approximately 55%, and that of metastatic RCC is approximately 10%^2^. However, at present biomarkers for early detection and follow-up of RCC are not available, which accounts for late diagnosis and subsequent poor prognosis. Therefore, searching for novel tumor biomarkers that enable the early detection and precise prognosis for patients with RCC has become a focus of basic and clinical RCC research.

MicroRNAs (miRNAs), a class of short noncoding RNA molecules, is recently discovered and shown to regulate gene expression at the post-transcriptional level, by binding through partial sequence homology, to the 3′ untranslated region (3′UTR) of mammalian target mRNAs and causing translational inhibition and/or mRNA degradation^3^. Mature miRNA which does not encode proteins, is incorporated into the RNA-induced silencing complex (RISC) and drives the selection of target mRNAs containing antisense sequences, thus controlling target gene expression^4^. MicroRNAs also function in tumor cell proliferation, apoptosis, invasion and tumor vessel formation via the mediation of downstream target genes. Moreover, it has been shown that miRNAs are aberrantly expressed or mutated in cancers, suggesting that miRNAs function as oncogenes or cancer suppressor genes in a tissue-specific manner^5^. Several studies have shown that aberrant miRNA expression is related to overall survival, disease stage and the development of metastases and recurrences. Recent evidence proposed miRNAs as promising biomarkers for early cancer detection and accurate prognosis as well as targets for more efficient treatment.

High-throughput technologies have been employed to identify differences in miRNA expression levels between tumor and normal tissues. Recently, increasing number of miRNA profiling datasets have grown rapidly, however, recent miRNA expression profiling datasets showed inconsistent results between the studies due to different technological platforms and small sample size application^6^,^7^,^8^,^9^,^10^,^11^,^12^,^13^,^14^,^15^,^16^,^17^,^18^,^19^,^20^,^21^,^22^,^23^,^24^,^25^,^26^,^27^,^28^,^29^,^30^,^31^,^32^,^33^,^34^. Considering inconsistent annotation and ongoing discovery of new miRNAs, different detection methods used by different technological platforms, various methods for data processing and analysis, we are trying to find a meaningful way in which to combine the results of several individual studies in order to increase the statistical power. Hence, we conducted an integrated miRNA expression profiling analysis which analysis datasets separately and then aggregate the resulting miRNA lists. This strategy has been applied to identify gene co-expression networks^35^ and to define more robust sets of cancer-related genes^36^ and miRNAs^37^,^38^.

These limitations prompted us to perform an integrated miRNA expression profiling analysis to (i) compare and validate the expression profiles of miRNA in RCC in comparison with normal tissue (NT), (ii) explore meta-signature miRNAs that consistently dysregulated expressed and bioinformatics function, (iii) identify possible associations between miRNA expression patterns and cancer specific survival (CSS), (iv) test the potential clinical usefulness of miRNAs as prognostic and predictive biomarkers and (v) provide a reliable meta-signature miRNAs based classifier that complements the traditional clinicopathological prognostic factors.

## Results

### Selection and a short overview of the datasets

A total of 13 renal cell carcinoma (RCC) miRNA expression profiling and 16 RCC miRNA expression datasets from 29 published studies were identified for this meta-analysis (Supporting Information Fig. 2). An expanded description of the design strategy is available in Supporting Information Fig. 1. First author, year of publication, acronym, country, period, assay type, disease, No. of probes, cancer type, clinical stage and No. of sample were extracted individually from each study and listed Supporting Information Table 1. A short description of the studies and the acronyms by which the studies are referred to in the following text is also provided in Supporting Information Table 1.

542 different expressed miRNAs were reported in the 29 miRNA expression profiling datasets; 11 were up-regulated (Supporting Information Table 2) and 38 were down-regulated (Supporting Information Table 3) in at least three studies. 10 and 9 studies reported relative miR-210 and miR-21 upregulated expression fold change, respectively. 14, 12 and 8 studies consistently reported relative miR-141, miR-200c and miR-429 downregulated expression fold change, respectively. Although there were differences between the individual miRNA profiling datasets, the top lists varied considerably from study to study.

**Figure 1:**
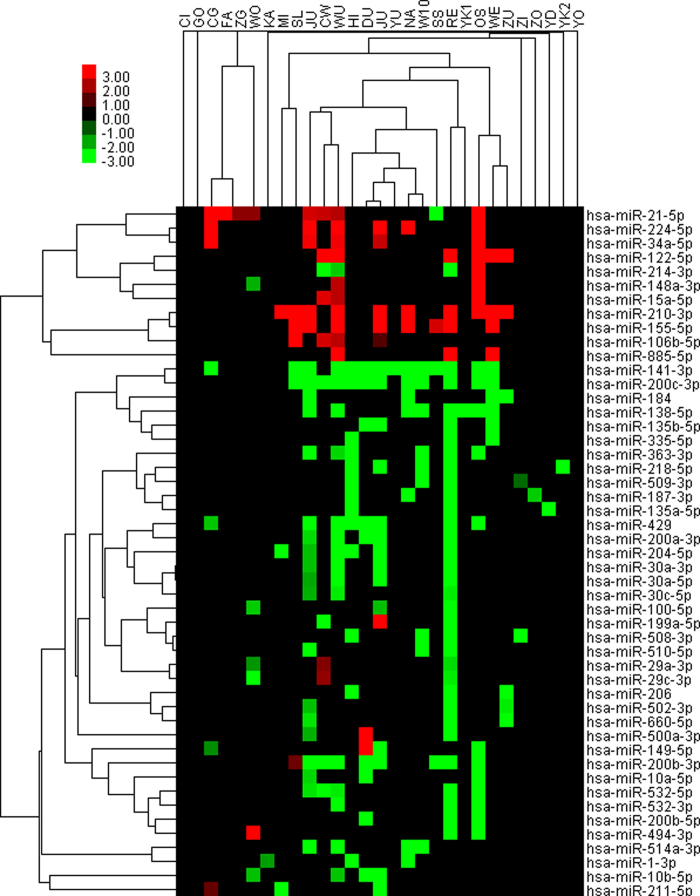
Heat map shows relative fold change of miRNAs in clear renal cell carcinoma compared with normal adjacent tissue as reported by eligible studies. Hierarchical clustering of 30 selected studies and datasets with the 49 deferentially expressed miRNAs using average linkage clustering. Here we selected 49 miRNAs (38 down-regulated miRNAs and 11up-regulated miRNAs) which reported in at least three expression profiling studies. Every row represents an individual miRNA, and each column represents an individual dataset. Acronyms are explained in Table 1 and the number of miRNAs analyzed in each study is graphically depicted on the right. Pseudocolours indicate transcript levels from low to high on a log 2 scale from –3 to 3, ranging from a low association strength (dark, black) to high (bright, red, or green).Short red and green vertical bars indicate upregulated and downregulated miRNAs, respectively. While the black bar with the pseudocolours 0 means that there is no available data reported in the primary studies.

To display the concordance of differentially miRNA profiling datasets more direct-viewing, heat-map and hierarchical clustering analysis were performed (Fig. 1). Short red and green vertical bars indicate upregulated and downregulated miRNAs, respectively. While the black bar with the pseudocolours 0 means that there is no available data reported in the primary studies. It shows that the results of the studies tend to cluster mainly according to utilized profiling platform^38^. It also clearly shows that most of the miRNA are consistently upregulated and downregulated. The five meta-signature miRNAs are the most reported, whereas only one study SS reported a reversed result of miR-21 expression in RCC.

### RCC miRNA meta-signature

We identified a statistically significant meta-signature of two upregulated and three downregulated miRNAs using robust rank aggregation (Table 1). Five of the most significantly dysregulated miRNAs, miR-21, miR-210, miR-141, miR-200c and miR-429 are reported by majority of the datasets (10, 9, 14, 12 and 8, respectively, Table 2). The direction of expression change of meta-signature miRNAs is consistent across all studies. The three meta-signature downregulated miRNAs (miR-141, miR-200c and miR-429) belong to the cluster of miR-200 family which plays an important in tumor epithelial-mesenchymal transition (EMT). Our results from the vote-counting strategy were almost the same with those from the Robust Rank Aggregation method. The distribution of miRNA rank scale is summarized in Supporting Information Fig. 3. We used three up-to-date prediction algorithms (TargetScan, miRanda and miRDB) to acquire conservative target predictions for the five meta-signature miRNAs. Gene set enrichment analysis found that meta-signature miRNAs cooperatively target functionally related and biologically relevant genes in signaling and developmental pathways. The top ten GO processes and pathways that were most strongly enriched with respect to the meta-signature miRNA candidates (miR-21, miR-210, miR-141, miR-200c and miR-429) are shown in Table 2.

### Experimental validation of the expression levels of the most dysregulated miRNAs in patients with clear renal cell carcinoma

Considering of the basis of the miRNA microarray results, we further examined renal-cancer-associated miRNA expression using qRT-PCR to analysis the 45 ccRCC samples in different sets so as to assess and validate the prognostic value of every candidate miRNA. RCC patients’ clinicopathological characters listed in Supporting Information Table 4. We selected top 15 dysregulated miRNAs for the validation qRT-PCR analysis. The results showed that the expression levels of miR-21, miR-210, miR-122, miR-155 and miR-224 were increased, whereas the levels of miR-138, miR-204, miR-218, miR-363, miR-532, miR-141, miR-200a, miR-200b, miR-200c and miR-429 were decreased in the RCC tissues compared with adjacent normal tissue (all *p* < 0.05). Detailed data are available in Supporting Information Fig. 4.

**Figure 2:**
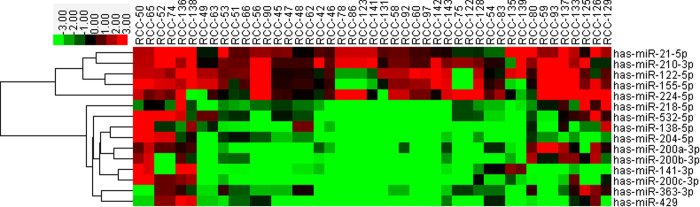
Heat map shows relative fold change of miRNAs in clear renal cell carcinoma compared with normal adjacent tissue determined by qRT-PCR. Hierarchical clustering of 45 paired tumor tissues and adjacent normal tissue with the 15 deferentially expressed miRNAs using Euclidean distance and average linkage clustering. Every row represents an individual gene, and each column represents an individual sample. Pseudocolours indicate transcript levels from low to high on a log 2 scale from –3 to 3, ranging from a low association strength (dark, black) to high (bright, red, or green).

To display the relative expression of differentially miRNA more direct-viewing, heat-map and hierarchical clustering analysis were also performed (Fig. 2). Short red and green vertical bars indicate upregulated and downregulated miRNAs, respectively. Using hierarchical clustering, based on the deferentially expressed miRNAs, successfully separated the 15 miRNAs into two discrete groups, upper five upregulated miRNAs and lower ten downregulated miRNAs (Fig. 2).

### Prognostic significance of the candidate meta-signature miRNAs in RCC

**Figure 3:**
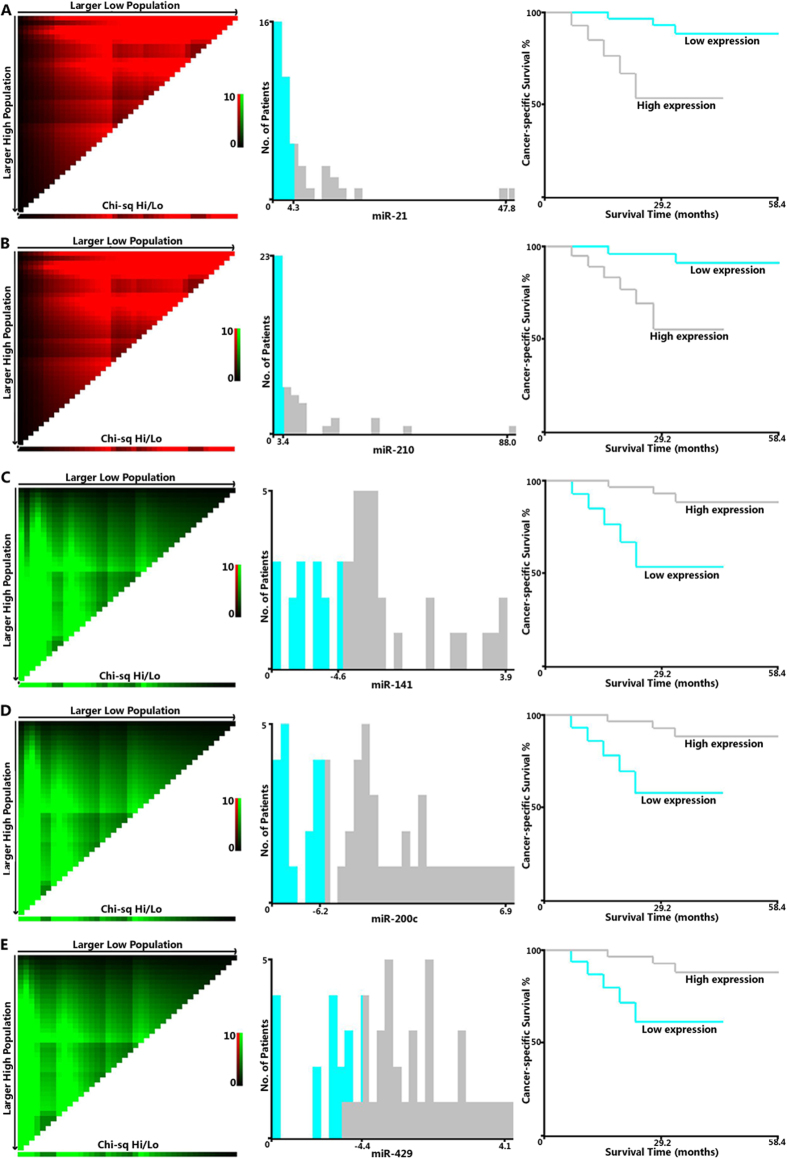
Kaplan-Meier cancer-specific survival analysis by X-tile plots cut-off point. X-tile plots of training sets are shown in the left panels. The plot showed the chi-squared log-rank values created when the cohort was divided into two groups .The optimal cut-point highlighted by the black circle in the left panels is shown on a histogram of the entire cohort (middle panels) and a Kaplan-Meier plot (right panels). P values were determined by using the cut-point defined in the training subset to parse a separate validation subset. The optimal cutpoint for miRNAs expression determined by X-tile analysis of the training cohort was applied to the validation cohort and reached high statistical significance (miR-21(A), miR-210(B), miR-141(C), miR-200c(D), miR-429(E)).

We used X-tile plots to generate the optimum cut-off point for the 15 differentially dysregulated miRNAs in the validation set (Fig. 3 and Supporting Information Fig. 5). We used Cox regression univariate analysis to build a prognostic classifier, which selected five miRNAs from the 15 miRNAs: miR-21 (HR: 5.46, 95%CI: 2.02-53.39), miR-210 (HR: 6.85, 95%CI: 2.13-43.36), miR-141 (HR: 0.12, 95%CI: 0.004-0.18), miR-200c (HR: 0.08, 95%CI: 0.01-0.43) and miR-429 (HR: 0.18, 95%CI: 0.02-0.50) (Table 3). However, there is no significant prognosis between the other ten miRNAs and RCC survival. Clinicopathologic factors including age, sex, BMI, tumor stage, grade, tumor size and metastasis, were also took into Cox regression analysis. High stage, low differentiation, large tumor size and distant metastasis had a poor cancer-specific survival (all *p* < 0.05; Table 3).

### Meta-analysis of signature miRNAs on predicting CSS in RCC

To determine if the five most deregulated miRNAs from the meta-signature analysis predicts CSS in RCC, we performed the meta-analysis of signature miRNAs as predictive biomarkers for RCC. A total of 4 studies reported miR-21 with CSS in patients with RCC. We extracted the hazard ratio on relative miRNAs expression and RCC CSS (Supporting Information Table 6 and Table 7). Our pooled results showed that upregulated miR-21 level was associated with poor CSS (HR: 1.96, 95% CI: 1.27-3.04; Supporting Information Fig. 6). There is no significant association between other dysregulated miRNAs (miR-210, miR-141, miR-200c; miR-429) and cancer-specific survival in RCC (Supporting Information Fig. 7, 8, 9; all *p* < 0.05).

### Identification of five meta-signature miRNAs based classifier by Cox regression analysis

Although X-tile and K-M survival analysis identified all the five meta-sinature miRNAs had prognostic significance, meta-analysis of signature miRNAs on predicting CSS in RCC showed that only miR-21 was significantly associated with poor survival. So, we considered single miRNA had limit in predicting RCC survival. We built a classifier based on the five miRNAs: miR-21, miR-210, miR-141, miR-200c and miR-429. We then derived the patients all dysregulated based on their individual five miRNA expression levels. Trying to divide patients with upregulated miR-21, miR-21 and simultaneously downregulated miR-141, miR-200c, miR-429 as the high-risk group, while the others are defined as low-risk group, we derived a formula to calculate the risk score, weighted by regression coincident based on their individual five miRNA expression levels. Using the Cox regression models, we calculated a risk score for each patient based on their individual expression levels of the five miRNAs, where risk score = (0.297×status of miR-21) + (0.189 × status of miR-210) - (0.221 × status of miR-141) - (0.186 × status of miR-200c) - (0.107 × status of miR-429). Multivariate Cox regression analysis showed our five meta-signature miRNAs based classifier was a reliable prognostic and predictive tool for disease recurrence in patients with RCC, especially in ccRCC (HR: 5.46, 95% CI: 1.51-19.66; Table 3).

## Discussion

There is increasing evidence to suggest that miRNA play an important role in predicting CSS in RCC, however, recent miRNA expression profiling datasets are lack of inconsistent results between the studies due to different technological platforms and lab protocols as well as small sample sizes application. Although the preferred method for miRNA expression meta-analysis involves analysis of the raw expression datasets that are pooled together, such rigorous approach is often not possible due to the unavailability of the raw data. Variations in the number of miRNAs known at the moment and the technological platform employed in any particular study make the proper integration of raw datasets very complicated. In addition, the relatively small sample size and noisiness of microarray data have resulted in inconsistency of biological conclusions. To overcome these limitations, it might be better to analysis datasets separately and then aggregate the resulting miRNA lists. In this study, we performed a meta-analysis approach to analyze RCC specific miRNAs derived from independent profiling datasets. Using robust rank aggregation method, we identified a meta-signature two upregulated and three downregulated miRNAs.

We suggest that the meta-signature miRNAs are key regulatory drivers in the oncogenesis. This is supported by the targeted gene enrichment analysis, which indicates very strong impact on several pathways related to p53, ErbB signaling pathway and pathways in cancer (Table 2). Therefore, these miRNAs may be good candidates for the development of tests for monitoring remission during postoperative follow-up. All of the meta-signature miRNAs are proven to be functionally important in RCC development. miR-21, one of the best-studied miRNAs, is upregulated in a variety of cancers. Our meta-analysis also displayed high level of miR-21 is associated with worse prognosis of RCC. Our another preparing paper showed miR-21 directly target the well-known tumor-suppressor PTEN. miR-210 is upregulated by HIF-1a in response to hypoxic conditions^39^. It has a number of validated targets, most notably associated with the regulation of mitochondrial metabolism but also with angiogenesis, apoptosis, cell cycle regulation and X-chromosome inactivation^40^. miR-141, miR-200c and miR-429 are all miR-200 family members as important epigenetic regulators of epithelial-mesenchymal transition (EMT) which have shown to provide significant prognostic value in a variety of cancers^41^,^42^.

We also highlight that a single miRNA had limit in predicting RCC survival. Although X-tile and K-M survival analysis identified all the five meta-sinature miRNAs had prognostic significance, meta-analysis of signature miRNAs on predicting CSS in RCC showed that only miR-21 was significantly associated with poor survival. Considering the limitation of single miRNA in RCC prognosis, we developed and validated a novel prognostic panel based on five-miRNAs to improve the prediction of CSS after surgery for patients with RCC. Furthermore, this proposed classifier can predict the survival of patients with RCC as well as tumor metastasis, and even better than other clinicopathological risk factors.

Recently, more and more studies focused to the unity of miRNAs either single miRNA or signature as the prognostic biomarkers in RCC. Of the five meta-signature miRNAs, miR-21 was the mostly used in various cancers, and demonstrated an exact effect for RCC prognosis^43^,^44^,^45^. Fresh-frozen tissues from 120 RCC, Silva-Santos *et al.*^44^ found miR-21, miR-141, and miR-155 convey prognostic information and might provide an ancillary tool for routine follow-up. McCormick *et al.*^46^ showed that high miR-210 expression is associated with better clinico-pathological prognostic factors. Validation with an independent 40-sample testing cohort of different stages of primary ccRCCs using the microarray data, Wu *et al.*^51^ identified a 4-microRNA signature for clear cell renal cell carcinoma metastasis and prognosis including miR-10b, miR-139-5p, miR-130b and miR-199b-5p. Based on 5 public miRNA expression datasets in ccRCC versus non-matching normal renal tissues from GEO database, Chen *et al.*^52^ revealed 11 clear cell renal cell carcinoma associated miRNA expression signatures (DE-miRNAs) including miR-210, miR-138, miR-16, miR-224, miR-34a, miR-184, miR-122, miR-126, miR-155, miR-15b and miR-660.

There are several factors that must be considered when choosing miRNAs as candidate prognostic biomarkers for RCC. First, the fold-change of the miRNA should be significant enough to discriminate tumor tissue from normal tissue. Second, the biological function and carcinogenesis mechanism of each individual miRNA should be thoroughly investigated in RCC. A better understanding of the targeted genes of the miRNAs would advance their use in clinical settings. As shown in Supporting Information Table 5, dysregulated miRNAs in RCC involved in cancer pathogenesis in other malignancies with universal biological function and targeted genes. Third, there should be adequate information about the expression of miRNAs in different types of specimens, that is to say we focused on studies that analysed miRNA expression between RCC tissues and normal tissue in humans. Fourth, rigorous validation and demonstration of reproducibility in an independent cohort of patients are necessary to confirm the prognostic value of miRNAs. Last but not least, a meta-analysis that combines current evidence with previous published data of individual miRNA associated CSS in RCC is necessary to confirm our conclusion of miRNA prognostic value. Although X-tile and K-M survival analyses identified all the five meta-sinature miRNAs had prognostic significance, meta-analysis of signature miRNAs on predicting CSS in RCC showed that only miR-21 was significantly associated with poor CSS. So, we considered single miRNA had limit in predicting RCC survival. Therefore, we built a classifier based on the five miRNAs: miR-21, miR-210, miR-141, miR-200c and miR-429.

To our knowledge, no meta-analysis of miRNA profiling studies has investigated RCC specially. Present meta-analysis has been proved to be useful in exploring candidate miRNA biomarkers in human RCC. The present study suggested five promising miRNAs that have been consistently reported with fold change. Their potential targets may provide some clues of miRNAs in tumorigenesis and the underlying mechanisms. However, we should admit that there exist certain inherent limitations in the studies included in our meta-analysis which cannot be ignored when interpreting our data. The major limitation of this study is the inconsistently results of eligible miRNA expression profiling datasets detected with different technological platforms. Second, our current study is limited because it is retrospective validation, with limited of small sample sizes as all patients are Chinese Clearly, our results should be further validated by prospective study in multicentre clinical trials. Third, our analysis is limited to comparison of tumor and normal tissue only. From the clinical point of view, it would be more interesting to explore non-invasive biomarkers associated with patient diagnosis, survival, disease aggressiveness or response to therapy. For this tissue resection based miRNA classfier would be just suitable for RCC prediction and prognosis after nephroectomy. Given on this, we are now identifying non-invasive circulating miRNAs in plasma which would be more cancer-specific for early detection and precise prediction. Fourth, several intensely investigated cancer-related miRNAs such as let-7, miR-10b cluster or mir-34 were not part of RCC meta-signature. Expression of these miRNAs has been specifically investigated in several studies^45^,^46^, but our meta-analysis suggests that their dysregulation fold change is relatively conservative. For example, miR-34a was found upregulated in all the six studies, but it did not reach the most statistical significance in our meta-analysis when all miRNAs were considered.

In summary, we performed a meta-analysis approach to critically converge and dissect quite heterogeneous miRNA expression profiling datasets in RCC. We identified a meta-signature, consisting of five highly significant and consistently dysregulated miRNAs (miR-21, miR-210, miR-141, miR-200c and miR-429) from 30 different datasets. These meta-signature miRNAs and gene interaction pathways affected by them are promising candidates for predictive and prognostic biomarkers in RCC. Further mechanistic and clinical validation studies are needed for their clinical significance and role in the development of RCC. Our analysis also highlights the challenges connected with the development of miRNA-based tests and emphasizes the need for rigorous evaluation of the results before proceeding to clinical trials.

## Material and Methods

### Selection of studies and datasets

#### Study selection

A systematic review of the literature was performed to identify renal cancer miRNA expression profiling studies published up to December 2013. We conducted a systematic search of the electronic databases, including Medline, Embase databases, and Cochrane library, using the MESH search headings: (mirna OR microrna OR mir-) AND profil AND (renal OR kidney) AND (cancer OR tumor OR carcinoma).

#### Inclusion criteria and exclusion criteria

To be included in the analysis, studies should meet the following criteria: (i), they were renal carcinoma miRNA expression profiling studies; (ii), they used tissue samples obtained from surgically resected renal carcinoma and corresponding adjacent or normal tissues; (iii), they reported the relative miRNAs expression via miRNA microarray or qRT-PCR.

Studies were excluded in the meta-analysis if: (i) the inclusion criteria were not met, (ii) no outcomes of interest were reported or were impossible to calculate or extrapolate the necessary data from the published results, (iii) studies using the serum, or sputum samples of renal carcinoma patients or cell lines, and (iv) reviews and the studies comparing miRNA expression profiles in metastatic and non-metastatic renal cell carcinoma were also excluded.

#### Data extraction

Two reviewers extracted independently the following data including: the first author, year of publication, country, study interval, assay type, No. of probes, cancer type, stage, No. of samples and the list of up- and down-regulated miRNA features, and their relative fold change. All disagreements about eligibility were resolved by a third reviewer by discussion until a consensus was reached.

### Bioinformatics analysis

#### Vote-counting rank

“Vote-counting rank” based sorting of potential molecular biomarkers was widely adopted in the meta-analysis^36^. miRNAs were ranked according to their importance as follows: (i) number of studies with consistent results; (ii) sample size in studies with consistent results; (iii) average fold-change in studies with consistent results. Total sample size was considered more important than average fold-change because many studies did not report the fold-change explicitly.

#### Robust rank aggregation analysis

All extracted miRNAs were ranked based on their associated p-values (less than 0.05 was considered significant) when their fold-changes were not reported. All of the protocols for the Robust Rank Aggregation method are free to download at the comprehensive R Archive Network website ( http://cran.r-project.org/). Details can be found in the package documentation ( http://cran.r-project.org/web/packages/RobustRankAggreg/RobustRankAggreg.pdf). This method assigns a p-value to each element in the aggregated list, which indicates how much better it is ranked compared with a null model, expecting random ordering^39^.

#### Cluster analysis of datasets

To assess the correlations between the results of individual studies, hierarchical cluster analysis was used. Overall rank matrix was constructed based on rank matrixes obtained from separate analyses for upregulated and downregulated gene lists. In the matrix, value 0.5 means that this miRNA was not reported in that study, value above 0.5 means it is upregulated (one minus normalized rank of miRNA from the analysis of upregulated gene lists) and value below 0.5 means that this miRNA is downregulated in that study (normalized rank from analysis of downregulated gene lists). In cluster analysis, Spearman rank correlation with average linkage method was used.

#### miRNA target prediction and enrichment analysis

The putative targets of meta-signature miRNAs were predicted using databases utilizing three different target prediction algorithms: TargetScan ( http://www.targetscan.org/), miRDB ( http://mirdb.org/miRDB/), and miRANDA ( http://www.microrna.org/microrna/getGeneForm.do), as each algorithm determines target binding differently. We selected targets from the miRANDA/miSVR search with scores less than −1.25 for further analysis. Enrichment analyses for KEGG and Panther pathways and Gene Ontology terms were performed with the GeneCodis tool ( http://genecodis.dacya.ucm.es/). The potential targets of each miRNA were used as input.

### Validation of the most up-regulated or down-regulated miRNAs using qRT-PCR

#### Sample collection

Forty-five ccRCC and adjacent normal tissue samples (collected post-operatively from Dec 2010 to Jan 2014) used in this study were obtained from the Department of Urology in Tongji Hospital of Huazhong University of Science and Technology (Wuhan, China). The specimens were obtained from patients undergoing ccRCC resection. All the diagnoses were based on pathology report. Upon removal of the surgical specimen, each sample was immediately frozen in liquid nitrogen and stored at −80 °C prior to RNA isolation and qRT-PCR analysis.

#### RNA extraction and qRT-PCR

Total RNA was isolated from the frozen tissue sample with TRIzol (Invitrogen) according to the manufacturer’s instructions. First-strand complementary DNA (cDNA) was synthesised from 2 μg of the total RNA using an oligo-dT primer and superscript II reverse transcriptase (Invitrogen). Then, quantification of the most up-regulated or down-regulated miRNAs was performed by qRT-PCR using SYBR Premix Ex Taq on MX3000 instrument. The U6 primers were obtained from GeneCopoeia. PCR was performed in a real-time PCR system as follows: 95 °C for 10 min, followed by 40 cycles of 95 °C for 10 sec, 60 °C for 20 sec and 72 °C for 30 sec, and then 95 °C for 1 min and 60 °C for 1 min. All experiments were done in triplicate. The expression level values were normalized to those of the small nuclear RNA U6 as a control. Relative fold-changes of miRNA expression were calculated using the ΔΔCT method, and the values were expressed as 2^−ΔΔCT^.

#### Follow-up

Patients’ follow-up after nephrectomy mainly consisted of routine physical examinations, medical imaging examinations. Medical imaging examinations, which included ultrasonography examinations, enhanced computerized tomography scan (CT) or positron emission tomography/computed tomography scans (PET/CT). All patients were followed retrospectively both through hospital records and by telephone interviews to patients and/or their close relatives. The length of follow-up was calculated from the date of surgery to the date of last clinical follow-up. All patients were followed up yearly, with the last follow-up being conducted in June 2014. All follow-up data were collected and analyzed. The spectrum of clinical follow-up included a history, physical examination, and routine biochemical profile, follow-up time, subsequent therapy of primary diseases and, if the patient died, the cause of death.

### Statistical analysis

#### Survival analysis

For survival analysis, we used the Kaplan-Meier method to analysis the correlation between variables and cancer-specific survival, and the log-rank test to compare survival curves. We used the Cox regression model to do the univariate and multivariate survival analysis. Kaplan-Meier survival analysis was used to analyze the association between postoperative CSS and the miRNA expression level measured by qRT-PCR, and the resulted curves were divided into two classes (high and low expression in comparison to the mean level of miRNA expression as the threshold). We selected the optimum cut-off score for the expression of every miRNA using X-tile plots based on the association with the patients’ CSS. X-tile plots provide a single and intuitive method to assess the association between variables and survival. The X-tile program can automatically select the optimum data cut point according to the highest χ^2^ value (minimum p value) defined by Kaplan–Meier survival analysis and log-rank test^40^. We did the X-tile plots using the X-tile software version 3.6.1 (Yale University School of Medicine, New Haven, CT, USA).

#### Meta-analysis

Hazard ratios (HRs) and 95% confidence intervals (CIs) were used to estimate the impact of miRNAs expression on CSS. A combined HR > 1 implied an unfavorable survival, and it was considered statistically significant if 95% CI for the combined HR did not overlap 1. We performed the meta-analysis by using the Review Manager Software (RevMan 5.1, Cochrane Collaboration, Oxford, UK).

## Author Contributions

TK carried out data analysis and drafted the manuscript; XH participated in study design, data collection and analysis. All authors read and approved the final manuscript.

## Additional Information

**How to cite this article**: Tang, K. and Xu, H. Prognostic value of meta-signature miRNAs in renal cell carcinoma: an integrated miRNA expression profiling analysis. *Sci. Rep.* **5**, 10272; doi: 10.1038/srep10272 (2015).

## Supplementary Material

Supplementary Information

## Figures and Tables

**Table 1:** RCC meta-signature miRNAs.

**miRNA name**	**Corrected p-value**	**Permutation p-value**	**No. of studies**
Up-regulated			
hsa-miR-210	2.46E-14	4.39E-16	10
hsa-miR-21	5.22E-11	6.34E-13	9
Down-regulated			
hsa-miR-141	2.58E-11	3.35E-13	14
hsa-miR-200c	1.18E-09	1.92E-11	12
hsa-miR-429	4.68E-08	5.28E-10	8

**Table 2:** Ten GO processes and pathways most strongly enriched by meta-signature miRNA targets.

GO processes	Process	P-Value	Benjamini	Genes
	0035556: intracellular signaling cascade	1.80E-11	7.80E-08	263
	0007167: enzyme linked receptor protein signaling pathway	1.10E-09	2.50E-06	92
	0019220: regulation of phosphate metabolic process	1.60E-09	2.30E-06	119
	0042325: regulation of phosphorylation	1.70E-08	1.90E-05	112
	0051338: regulation of transferase activity	2.20E-08	1.90E-05	94
	0043549: regulation of kinase activity	5.00E-08	3.60E-05	90
	0031328: positive regulation of cellular biosynthetic process	2.10E-07	1.10E-04	147
	0045859: regulation of protein kinase activity	3.50E-07	1.50E-04	85
	0045893: positive regulation of transcription, DNA-dependent	2.40E-06	5.30E-04	106
	0006357: regulation of transcription from RNA polymerase II promoter	2.80E-06	5.60E-04	149
KEGG Pathways	Pathway	P-Value	Benjamini	Genes
	04722: p53 signaling pathway	1.50E-06	1.40E-04	27
	05200: Pathways in cancer	2.70E-06	1.70E-04	80
	04510: Focal adhesion	3.90E-06	1.80E-04	55
	04012: ErbB signaling pathway	9.50E-06	3.50E-04	30
	04910: Insulin signaling pathway	1.50E-05	4.50E-04	40
	04310: Wnt signaling pathway	4.50E-05	1.20E-03	42
	04520: Adherens junction	6.40E-05	1.50E-03	26
	05220: Chronic myeloid leukemia	1.20E-04	2.40E-03	25
	04010: MAPK signaling pathway	3.60E-04	5.60E-03	61
	04350: TGF-beta signaling pathway	5.50E-04	7.80E-03	26
Panther pathways	Pathway	P-Value	Benjamini	Genes
	P00039: Metabotropic glutamate receptor group III pathway	2.20E-04	2.70E-02	26
	P00059: p53 pathway	2.60E-04	1.60E-02	35
	P04398: p53 pathway feedback loops 2	8.50E-04	3.50E-02	21
	P00034: Integrin signalling pathway	2.80E-03	8.50E-02	51
	P00052: TGF-beta signaling pathway	3.60E-03	8.70E-02	38
	P00018: EGF receptor signaling pathway	5.50E-03	1.10E-01	35
	P04391: Oxytocin receptor mediated signaling pathway	7.60E-03	1.30E-01	18
	P04394: Thyrotropin-releasing hormone receptor signaling pathway	9.10E-03	1.30E-01	18
	P04374: 5HT2 type receptor mediated signaling pathway	1.50E-02	1.90E-01	19
	P04378: Beta2 adrenergic receptor signaling pathway	1.80E-02	2.00E-01	14

The number of predicted target genes in the process or pathway is shown.

**Table 3:** Univariate and multivariate Cox regression analysis of miRNAs and clinicopathologic parameters in clear cell renal cell carcinoma (ccRCC) patients in relation to cancer-specific survival.

**Variables (and stratification)**	**Univariate analysis**		**Multivariate analysis**	
	**HR (95% CI)**	***p*****-value**	**HR (95% CI)**	***p*****-value**
Age (>60 years)	1.10 (0.46 - 2.66)	0.814	/	/
Sex (male/female)	0.50 (0.17 - 1.49)	0.188	/	/
BMI (>25.51 kg/m^2^)	1.08 (0.58 - 2.02)	0.81	/	/
Tumor stage (T3-4/T1-2)	1.05 (1.01 - 1.09)	0.036	1.18 (0.80 - 1.73)	0.4
Tumor grade (G3/G1-2)	1.09 (1.05 - 1.14)	0.0002	1.06 (0.99 - 1.13)	0.11
Tumor size (>55 mm)	5.64 (2.04 - 15.7)	0.0003	3.51 (1.13 - 10.94)	0.03
Metastases (yes/no)	17.6 (8.89 - 34.9)	<0.0001	7.29 (1.85- 28.73)	0.005
hsa-miR-21 (high *vs.* low)	5.46 (2.02 - 53.39)	0.0068	6.46 (1.35 - 30.94)	0.02
hsa-miR-210 (high *vs.* low)	6.85 (2.13 - 43.36)	0.0045	3.27 (1.01 - 10.59)	0.05
hsa-miR-122 (high *vs.* low)	3.37 (0.90 - 17.07)	0.073	/	/
hsa-miR-155 (high *vs.* low)	3.58 (0.78 -16.36)	0.0997	/	/
hsa-miR-224 (high *vs.* low)	1.46 (0.35 - 6.20)	0.606	/	/
hsa-miR-138 (high *vs.* low)	0.28 (0.05 - 0.99)	0.056	/	/
hsa-miR-204 (high *vs.* low)	0.37 (0.08 - 1.36)	0.137	/	/
hsa-miR-218 (high *vs.* low)	0.55 (0.12 - 2.17)	0.378	/	/
hsa-miR-363 (high *vs.* low)	0.30 (0.06 - 1.08)	0.0715	/	/
hsa-miR-532 (high *vs.* low)	0.49 (0.10 - 1.93)	0.2915	/	/
hsa-miR-141 (high *vs.* low)	0.12 (0.004 - 0.18)	0.0003	0.25 (0.08 - 0.86)	0.03
hsa-miR-200a (high *vs.* low)	0.45 (0.08 - 1.55)	0.1963	/	/
hsa-miR-200b (high *vs.* low)	0.34 (0.07 - 1.24)	0.1084	/	/
hsa-miR-200c (high *vs.* low)	0.08 (0.01 - 0.43)	0.0036	0.31 (0.09 - 1.05)	0.06
hsa-miR-429 (high *vs.* low)	0.18 (0.02 - 0.50)	0.0068	0.51 (0.1 - 2.32)	0.39
Meta-signature miRNAs	3.30 (1.94 - 5.63)	<0.0001	5.46 (1.51 - 19.66)	0.009

hsa-miR, Homo sapiens microRNA; T, tumor classification; G, histopathological grading; BMI, body mass index.

Meta-signature miRNAs, five RCC specified miRNA (miR-21, miR-210, miR-141, miR-200c, miR-429) based classifier (high risk vs. low risk).

All 45 RCC patients dichotomized in relation to the indicated stratification criteria were included in the Cox regression analysis for clinicopathological factors.
