# Supplementary material for: Prognostic value of meta-signature miRNAs in renal cell carcinoma: an integrated miRNA expression profiling analysis
Source: Sci Rep. 2015 May 14;5:10272. doi: 10.1038/srep10272 (PMC4431463; doi:10.1038/srep10272)
Supplement: Supplementary Information [file srep10272-s1.doc]

**Title page**

Title:

**Prognostic value of meta-signature miRNAs in renal cell carcinoma: an integrated miRNA expression profiling analysis**

Authors: Kun Tang ^1,2^, Hua Xu ^1,2*^

1, Department of Urology, Tongji Hospital, Tongji Medical College, Huazhong University of Science and Technology, Wuhan, China

2, Institute of Urology, Tongji Hospital, Tongji Medical College, Huazhong University of Science and Technology, Wuhan, China

*To whom correspondence should be addressed at: Prof. Hua Xu

Department of Urology, Tongji Hospital, Tongji Medical College, Huazhong University of Science and Technology, Wuhan 430030, China.

Phone: 86-27-836-63454; Fax: 86-27-836-63454, Email: [xuhuawhu@163.com](mailto:xuhuawhu@163.com)

**Key words:** renal cell carcinoma, miRNA proﬁles, miRNA signature, prognosis, integrated analysis

This manuscript includes 17 pages, 3 figures, 3 tables. Other 10 figures and 6 tables showed in supporting information.

**Supplementary information**

**Supplemental Figure S1** Overview of the design strategy.

**Supplemental Figure S2** Flow chart of studies identified, included, and excluded.

**Supplemental Figure S3** The size of deregulated miRNA lists varies greatly across the studies. Vertical boxes designate the number of signiﬁcantly upregulated (a) or downregulated (b) miRNAs. The rank scale is shown above. Positions of RCC meta-signature miRNAs, identiﬁed by robust rank aggregation analysis are highlighted.

**Supplemental Figure S4** Relative expression of miRNAs in clear renal cell carcinoma compared with normal adjacent tissue determined by qRT-PCR (miR-21(A), miR-210(B), miR-122(C), miR-155(D), miR-224(E), miR-138(F), miR-204(G), miR-218(H), miR-363(I), miR-532(J), miR-141(K), miR-200a(L), miR-200b(M), miR-200c(N), miR-429(O)).

**Supplemental Figure S5** Kaplan-Meier cancer-speciﬁc survival analysis by X-tile plots cut-off point. X-tile plots of training sets are shown in the left panels. The plot showed the chi-squared log-rank values created when the cohort was divided into two groups .The optimal cut-point highlighted by the black circle in the left panels is shown on a histogram of the entire cohort (middle panels) and a Kaplan-Meier plot (right panels). P values were determined by using the cut-point defined in the training subset to parse a separate validation subset. The optimal cutpoint for miRNAs expression determined by X-tile analysis of the training cohort was applied to the validation cohort and reached high statistical signiﬁcance (miR-122(A), miR-155(B), miR-224(C), miR-138(D), miR-200a(E), miR-200b(F), miR-204(G), miR-218(H), miR-363(I), miR-532(J)).

**Supplemental Figure S6** Forest plots of hazard ratios for miR-21 relative expression in patients with clear renal cell carcinoma (CRCC) cancer-specific survival (CSS).

**Supplemental Figure S7** Forest plots of hazard ratios for miR-210 relative expression in patients with clear renal cell carcinoma (CRCC) cancer-specific survival (CSS).

**Supplemental Figure S8** Forest plots of hazard ratios for miR-141 relative expression in patients with clear renal cell carcinoma (CRCC) cancer-specific survival (CSS).

**Supplemental Figure S9** Forest plots of hazard ratios for miR-200c relative expression in patients with clear renal cell carcinoma (CRCC) cancer-specific survival (CSS).

**Supplemental Figure S10** Forest plots of hazard ratios for miR-429 relative expression in patients with clear renal cell carcinoma (CRCC) cancer-specific survival (CSS).

**Supplemental Table S1** Characteristics of analyzed datasets.

**Supplemental Table S2** Up-regulated miRNAs (n=11) reported in at least three expression profiling studies.

**Supplemental Table S3** Down-regulated miRNAs (n=38) reported in at least three expression profiling studies.

**Supplemental Table S4** Patients’ clinicopathological characters.

**Supplemental Table** **S5** miRNAs dysregulated in renal cell carcinoma and their involvement in cancer pathogenesis in other malignancies.

**Supplemental Table** **S6** Estimation of the hazard ratio on relative miRNAs expression and renal cell carcinoma cancer-specific survival.

**Table** **S7 Pooling data from eligible studies included for meta-analysis which reported prognosis of the five meta-signature miRNAs in RCC**

Fig. S1


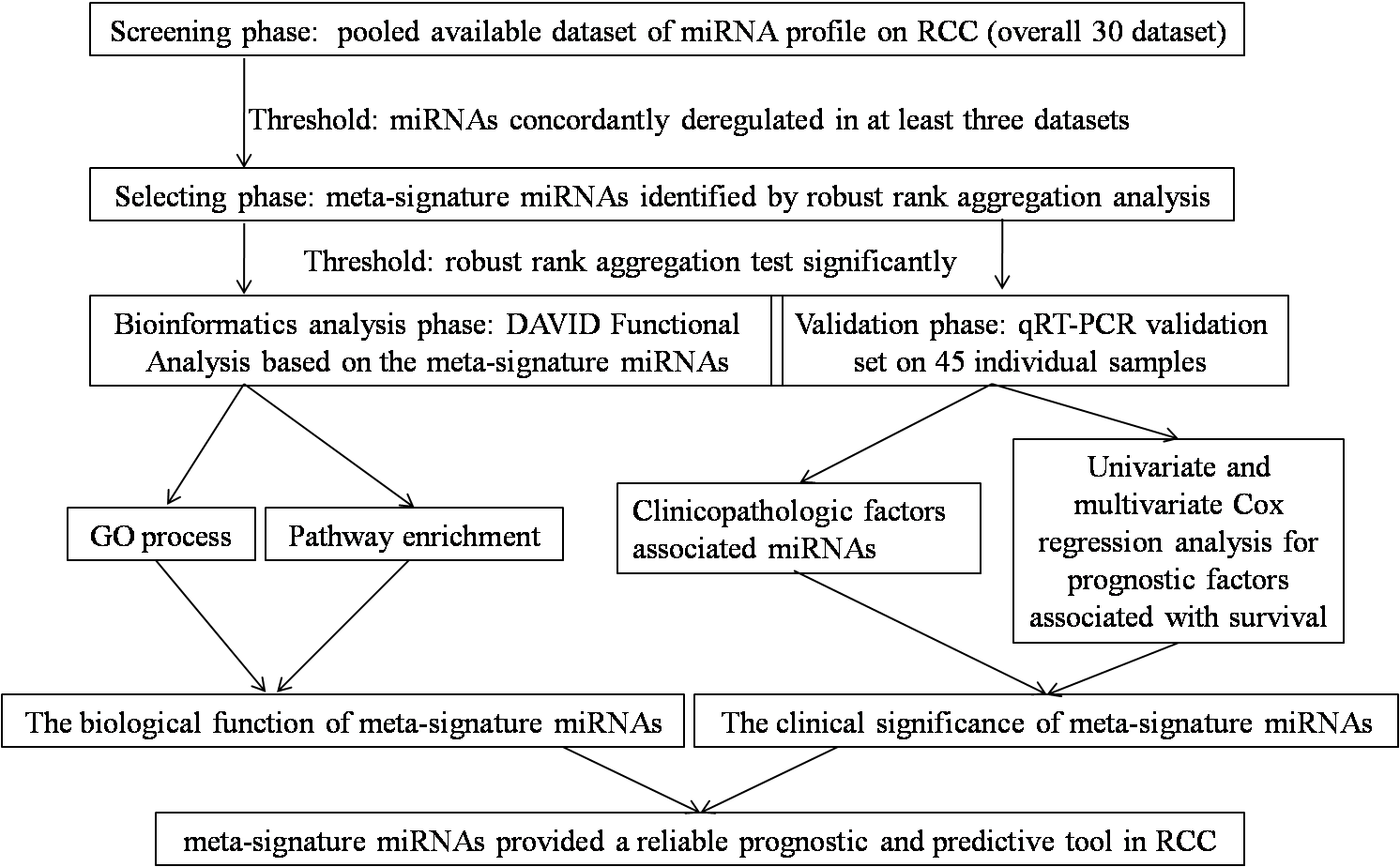


Fig. S2


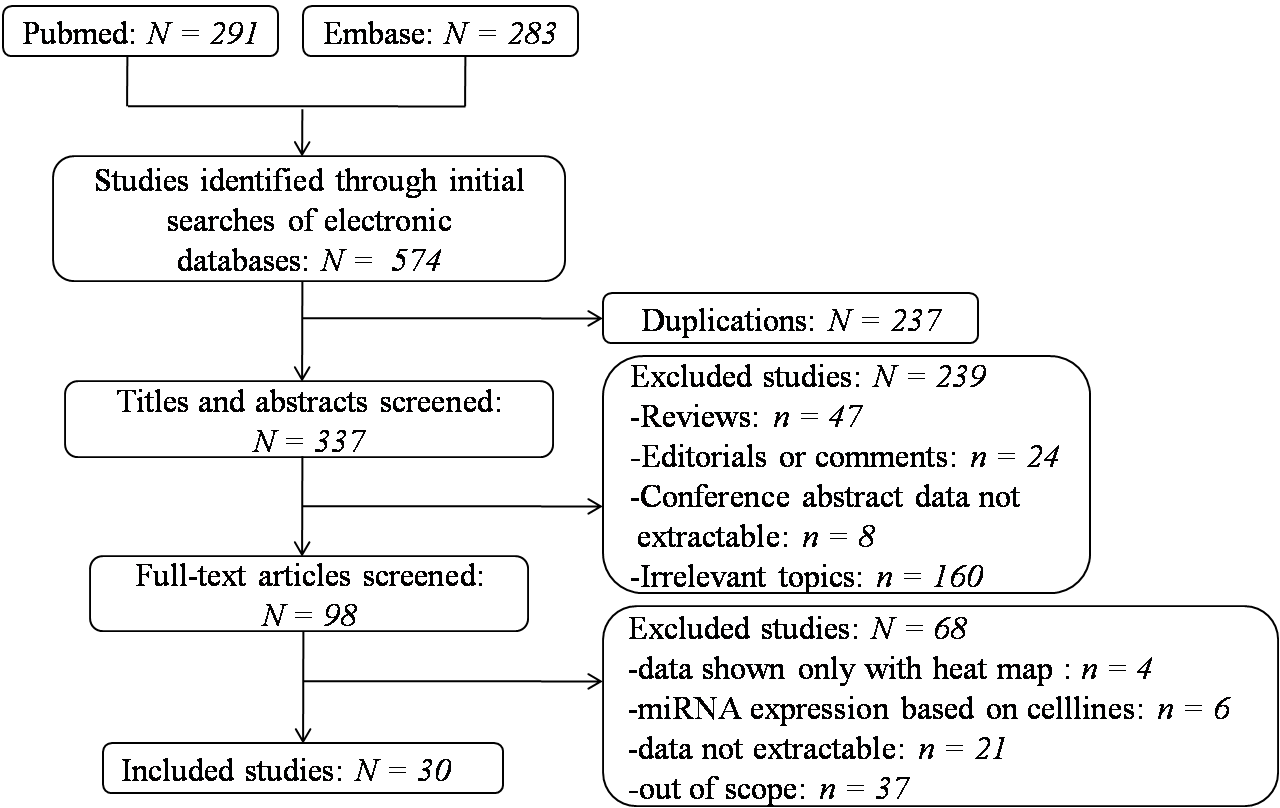


Fig. S3a


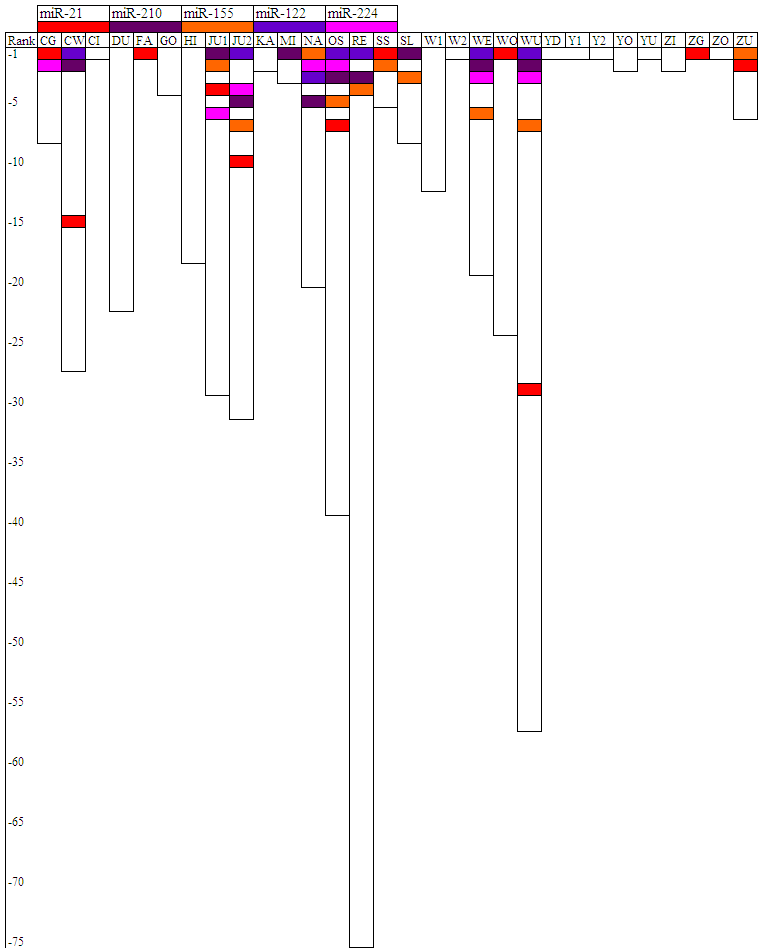


Fig.S3b


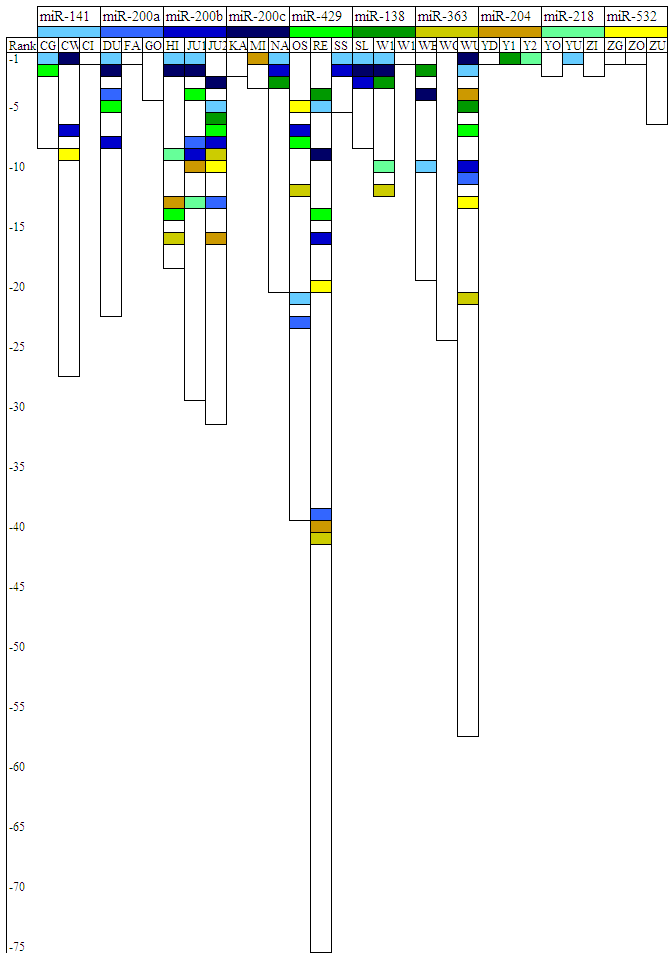


Fig. S4


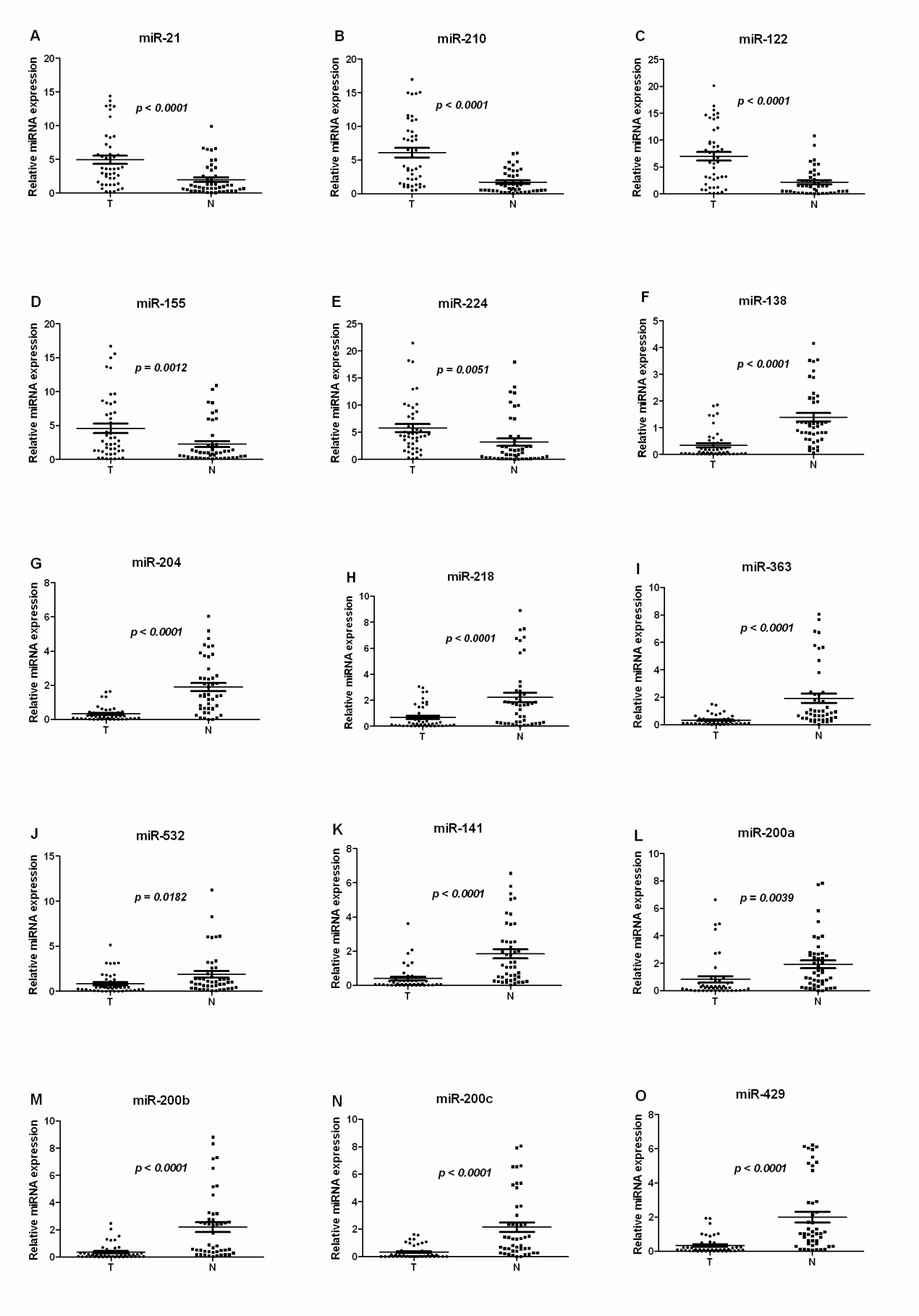


**Supplemental Figure S5**

**
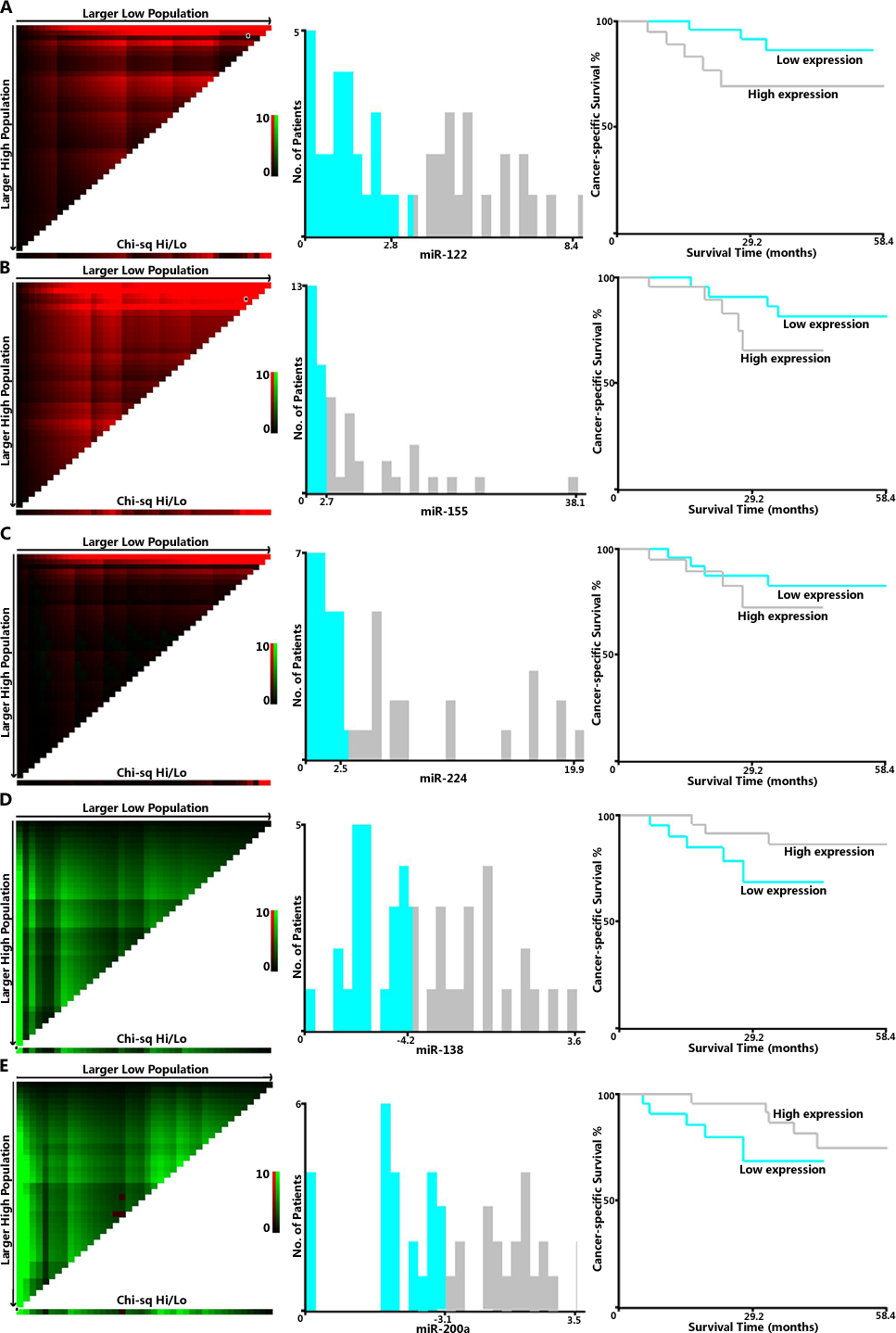
**

**Supplemental Figure S5 (continued)**

**
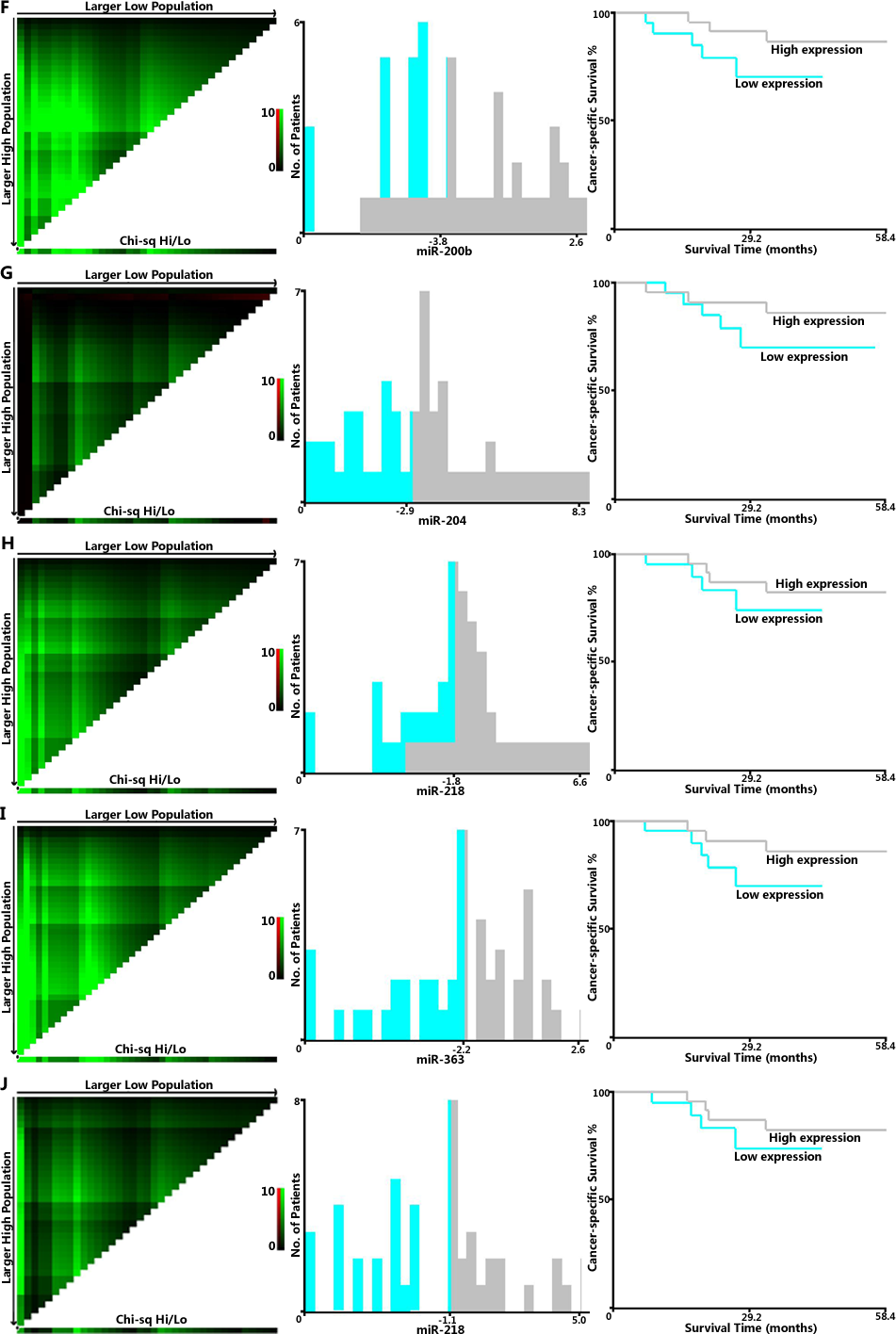
**

**Supplemental Figure S6** Forest plots of hazard ratios for miR-21 relative expression in patients with clear renal cell carcinoma (CRCC) cancer-specific survival (CSS).

**Supplemental Figure S7** Forest plots of hazard ratios for miR-210 relative expression in patients with clear renal cell carcinoma (CRCC) cancer-specific survival (CSS).

**
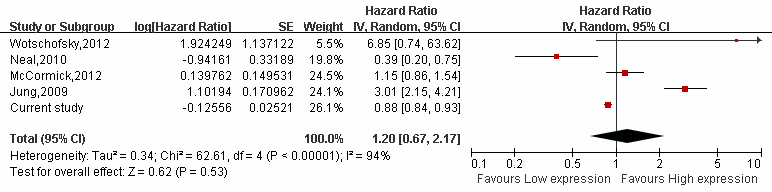
**

**Supplemental Figure S8** Forest plots of hazard ratios for miR-141 relative expression in patients with clear renal cell carcinoma (CRCC) cancer-specific survival (CSS).

**
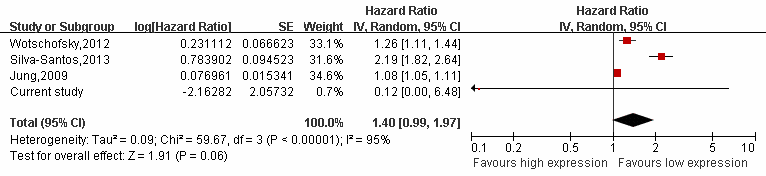
**

**Supplemental Figure S9** Forest plots of hazard ratios for miR-200c relative expression in patients with clear renal cell carcinoma (CRCC) cancer-specific survival (CSS).

**
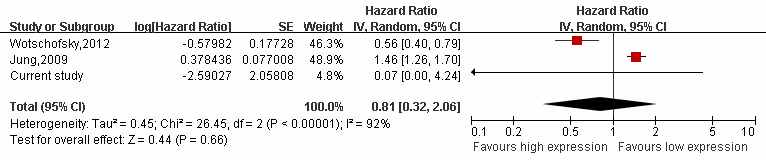
**

**Supplemental Figure S10** Forest plots of hazard ratios for miR-429 relative expression in patients with clear renal cell carcinoma (CRCC) cancer-specific survival (CSS).


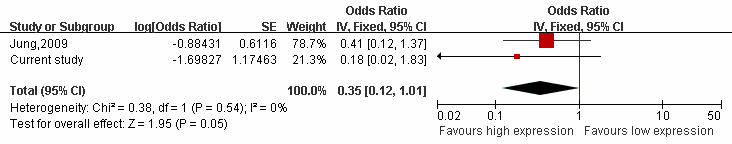


**Table S1.** Characteristics of analyzed datasets

| Reference | Acronym | Region | Period | Assay type | No. of probes | Cancer type | Clinical Stage | No. of samples |
| --- | --- | --- | --- | --- | --- | --- | --- | --- |
| Cheng,2013 ^6^ | CG | China | 2010-2011 | qRT-PCR | 8 | ccRCC | T1a-3b | 30(15/15) |
| Chow,2010 ^7^ | CW | Canada | NA | ulParaflo microfluidic chip | 80 | ccRCC | NA | 6(3/3) |
| Cui,2012 ^8^ | CI | China | 2005-2007 | qRT-PCR | 1 | ccRCC | T1a-3b | 80(40/40) |
| Duns,2013 ^9^ | DU | Netherlands | NA | Agilent Human miRNA v2 | 542 | ccRCC | NA | 12(10/2) |
| Faragalla,2012 ^10^ | FA | Canada | NA | qRT-PCR | 1 | ccRCC | T1a-3b | 85(71/14) |
| Gottardo,2007 ^11^ | GO | USA | 1998-2000 | PerkinElmer ScanArray XL5K | 76 | RCC | T1-4 | 23(20/3) |
| Hidaka,2012 ^12^ | HI | Japan | 2006-2009 | qRT-PCR | 103 | RCC | NA | 15(10/5) |
| Juan,2010 ^13^ | JU | USA | NA | 96-Well qPCR array | 35 | ccRCC | NA | 20(10/10) |
| Jung,2009 ^14^ | JU | Germany | 2004-2008 | Agilent Human miRNA Array | 534 | ccRCC | T1a-3b | 24(12/12) |
| Kawakami,2012 ^15^ | KA | Japan | NA | qRT-PCR | 2 | ccRCC | T1a-4 | 80(40/40) |
| Mikhaylova,2012 ^16^ | MI | Japan | NA | qRT-PCR | 3 | ccRCC | NA | 242(128/114) |
| Nakada,2008 ^17^ | NA | Japan | NA | G4470A Human MiRNA Microarray | 534 | ccRCC | T1a-3b | 64(32/32) |
| Osanto,2012 ^18^ | OS | Netherlands | 1997-2003 | Next Generation Deep Sequencing | 463 | ccRCC | T1a-3b | 22(11/11) |
| Redova,2013 ^19^ | RE | Czech Republic | 2003-2008 | TaqMan MicroRNA Array | 54 | RCC | T1a-3b | 59(59/59) |
| Silva-Santos,2013 ^20^ | SS | Portugal | 2003-2007 | qRT-PCR | 5 | ccRCC | T1a-4 | 130(120/10) |
| Slaby,2010 ^21^ | SL | Czech Republic | 2003-2009 | qRT-PCR | 8 | RCC | T1a-3b | 48(38/10) |
| Wang1,2013 ^22^ | W10 | China | NA | Agilent Human miRNA Array | 961 | RCC | NA | 10(5/5) |
| Wang2,2013 ^23^ | W11 | China | 2002-2009 | qRT-PCR | 1 | RCC | T1a-4 | 96/96 |
| Weng,2010 ^24^ | WE | USA | NA | MicroRNA Human Version 2 | 723 | ccRCC | T1a-3b | 6(3/3) |
| Wotschofsky,2012 ^25^ | WO | Germany | 2003-2010 | Agilent microarray chip | 470 | RCC | T1a-3b | 44(22/22) |
| Wu,2012 ^26^ | WU | USA | 1986-2008 | Agilent microarray chip | 723 | ccRCC | T1a-4 | 38(28/10) |
| Yamada,2012 ^27^ | YD | Japan | 2006-2009 | qRT-PCR | 1 | ccRCC | T1a-3b | 76(38/38) |
| Yamasaki,2012 ^28^ | YK1 | Japan | NA | qRT-PCR | 1 | RCC | T1a-3b | 66(33/33) |
| Yoshino,2013 ^29^ | YO | Japan | NA | TaqMan MicroRNA Array | 1 | RCC | NA | 36(18/18) |
| Yu,2013 ^30^ | YU | China | NA | qRT-PCR | 1 | RCC | NA | 40(20/20) |
| Zhai,2012 ^31^ | ZI | China | NA | qRT-PCR | 2 | RCC | T1a-4 | 20(10/10) |
| Zhang,2011 ^32^ | ZG | China | NA | qRT-PCR | 1 | RCC | NA | 60(40/20) |
| Zhao,2013 ^33^ | ZO | China | NA | qRT-PCR | 1 | ccRCC | T1a-4 | 108(54/54) |
| Zhou,2010 ^34^ | ZU | China | NA | qRT-PCR | 6 | ccRCC | NA | 20(10/10) |
| RCC, renal cell carcinoma; ccRCC, clear cell RCC; qRT-PCR, quantitative real-time polymerase chain reaction;NA, data not available. | | | | | | | | |

Table S2 Up-regulated miRNAs (n=11) reported in at least three expression profiling studies

| miRNA | Study reference | No. | Fold-change | | | | | | | | | |
| --- | --- | --- | --- | --- | --- | --- | --- | --- | --- | --- | --- | --- |
| has-miR-210-3p | 8,9,11,12,13,14,16,19,21,30 | 10 | 10.15 | 4.30 | 7.54 | 13.47 | 14.43 | 5.99 | 65.79 | 10.41 | 11.39 | 6.96 |
| has-miR-21-5p | 1,2,5,9,13,15,20,21,28 | 9 | 30.79 | 2.31 | 10.50 | 2.50 | 3.19 | 21.37 | 1.68 | 2.14 | 1.67 |  |
| has-miR-155-5p | 8,9,12,14,15,16,19,21 | 8 | 7.13 | 3.20 | 6.07 | 4.57 | 2.51 | 28.51 | 7.16 | 4.17 |  |  |
| has-miR-122-5p | 2,13,14,19,21,30 | 6 | 22.32 | 332.48 | 43.35 | 172.45 | 14.62 | 32.00 |  |  |  |  |
| has-miR-224-5p | 1,8,9,12,13,21 | 6 | 22.17 | 3.79 | 4.40 | 6.28 | 14.46 | 7.78 |  |  |  |  |
| has-miR-34a-5p | 1,8,9,13,21 | 5 | 6.40 | 2.34 | 2.90 | 4.24 | 2.85 |  |  |  |  |  |
| has-miR-106b-5p | 2,8,16,21 | 4 | 2.51 | 1.00 | 4.80 | 2.22 |  |  |  |  |  |  |
| has-miR-148a-3p | 13,20,21 | 3 | 3.04 | 2.10 | 2.19 |  |  |  |  |  |  |  |
| has-miR-15a-5p | 2,13,21 | 3 | 2.66 | 3.04 | 2.22 |  |  |  |  |  |  |  |
| has-miR-211-5p | 1,8,11 | 3 | 1.21 | 4.00 | 6.84 |  |  |  |  |  |  |  |
| has-miR-885-5p | 14,19,21 | 3 | 21.38 | 9.19 | 5.10 |  |  |  |  |  |  |  |
| miRNA expression fold-change upregulated are in black and downregulated are in red | | | | | | | | | | | | |

Table S3 Down-regulated miRNAs (n=38) reported in at least three expression profiling studies

| miRNA | Study reference | No. | Fold-change | | | | | | | | | | | | | |
| --- | --- | --- | --- | --- | --- | --- | --- | --- | --- | --- | --- | --- | --- | --- | --- | --- |
| has-miR-141-3p | 1,4,7,8,9,12,13,14,15,16,17,19,21,26 | 14 | 5.2 | 73.8 | 47.6 | 100.0 | 7.3 | 100 | 3.4 | 71.4 | 31.0 | 18.1 | 104.0 | 66.3 | 17.1 | 5.0 |
| has-miR-200c-3p | 2,4,7,8,9,12,13,14,16,17,19,21 | 12 | 39.7 | 40.4 | 47.6 | 50.0 | 8.8 | 98.0 | 3.2 | 76.9 | 9.0 | 99.8 | 86.2 | 17.4 |  |  |
| has-miR-200b-3p | 2,4,8,9,13,14,15,16,21 | 9 | 4.3 | 6.7 | 4.3 | 3.1 | 7.4 | 7.0 | 13.4 | 1.3 | 3.6 |  |  |  |  |  |
| has-miR-138-5p | 9,12,13,14,17,19,21,23 | 8 | 5.2 | 49.0 | 3.2 | 104.2 | 22.8 | 77.7 | 5.6 | 6.3 |  |  |  |  |  |  |
| has-miR-429 | 1,4,7,8,9,13,14,21 | 8 | 2.3 | 9.3 | 5.7 | 8.3 | 3.4 | 5.1 | 9.5 | 4.2 |  |  |  |  |  |  |
| has-miR-204-5p | 7,8,9,11,14,21 | 6 | 5.8 | 4.3 | 2.3 | 7.7 | 7.6 | 6.8 |  |  |  |  |  |  |  |  |
| has-miR-363-3p | 7,9,13,14,17,21 | 6 | 5.6 | 3.0 | 3.7 | 4.8 | 4.5 | 2.5 |  |  |  |  |  |  |  |  |
| has-miR-200a-3p | 4,8,9,14,21 | 5 | 10.2 | 4.3 | 2.6 | 6.5 | 3.3 |  |  |  |  |  |  |  |  |  |
| has-miR-218-5p | 7,8,14,17,24 | 5 | 7.7 | 3.8 | 6.0 | 5.1 | 4.7 |  |  |  |  |  |  |  |  |  |
| has-miR-532-5p | 2,9,13,14,21 | 5 | 3.7 | 2.7 | 7.8 | 5.7 | 2.8 |  |  |  |  |  |  |  |  |  |
| has-miR-10b-5p | 4,8,20,21 | 4 | 6.5 | 3.6 | 2.3 | 2.3 |  |  |  |  |  |  |  |  |  |  |
| has-miR-100-5p | 8,14,18,20 | 4 | 2.3 | 6.5 | 2.8 | 2.4 |  |  |  |  |  |  |  |  |  |  |
| has-miR-135b-5p | 4,8,14,19 | 4 | 6.2 | 3.4 | 6.5 | 11.5 |  |  |  |  |  |  |  |  |  |  |
| has-miR-149-5p | 1,4,8,13 | 4 | 1.7 | 3.0 | 3.3 | 3.6 |  |  |  |  |  |  |  |  |  |  |
| has-miR-184 | 9,12,19,30 | 4 | 12.1 | 16.3 | 129.8 | 51.3 |  |  |  |  |  |  |  |  |  |  |
| has-miR-187-3p | 7,12,14,29 | 4 | 35.7 | 15.6 | 36.1 | 2.4 |  |  |  |  |  |  |  |  |  |  |
| has-miR-214-3p | 2,13,14,21 | 4 | 5.5 | 53.3 | 4.1 | 2.4 |  |  |  |  |  |  |  |  |  |  |
| has-miR-30a-3p | 8,9,14,21 | 4 | 3.4 | 2.2 | 4.4 | 3.6 |  |  |  |  |  |  |  |  |  |  |
| has-miR-30a-5p | 8,9,14,21 | 4 | 3.8 | 2.0 | 4.7 | 4.5 |  |  |  |  |  |  |  |  |  |  |
| has-miR-508-3p | 7,14,17,27 | 4 | 13.9 | 144.9 | 7.8 | 6.7 |  |  |  |  |  |  |  |  |  |  |
| has-miR-509-3p | 7,14,17,27 | 4 | 23.8 | 11.3 | 8.7 | 1.3 |  |  |  |  |  |  |  |  |  |  |
| has-miR-514a-3p | 9,12,17,21 | 4 | 9.5 | 53.0 | 18.4 | 7.9 |  |  |  |  |  |  |  |  |  |  |
| has-miR-1-3p | 7,10,12 | 3 | 5.4 | 1.9 | 15.0 |  |  |  |  |  |  |  |  |  |  |  |
| has-miR-10a-5p | 4,9,13 | 3 | 6.3 | 2.5 | 10.8 |  |  |  |  |  |  |  |  |  |  |  |
| has-miR-135a-5p | 7,14,22 | 3 | 15.4 | 30.3 | 5.2 |  |  |  |  |  |  |  |  |  |  |  |
| has-miR-199a-5p | 2,8,14 | 3 | 3.7 | 3.3 | 12.6 |  |  |  |  |  |  |  |  |  |  |  |
| has-miR-200b-5p | 4,13,14 | 3 | 3.7 | 5.0 | 3.3 |  |  |  |  |  |  |  |  |  |  |  |
| has-miR-206 | 7,14,30 | 3 | 8.3 | 10.6 | 16.0 |  |  |  |  |  |  |  |  |  |  |  |
| has-miR-29a-3p | 2,14,20 | 3 | 1.5 | 2.6 | 1.8 |  |  |  |  |  |  |  |  |  |  |  |
| has-miR-29c-3p | 2,14,20 | 3 | 1.7 | 2.8 | 3.6 |  |  |  |  |  |  |  |  |  |  |  |
| has-miR-30c-3p | 9,14,21 | 3 | 2.1 | 2.9 | 2.8 |  |  |  |  |  |  |  |  |  |  |  |
| has-miR-335-5p | 7,14,19 | 3 | 5.5 | 4.8 | 10.9 |  |  |  |  |  |  |  |  |  |  |  |
| has-miR-494-3p | 13,14,20 | 3 | 15.1 | 8.2 | 5.7 |  |  |  |  |  |  |  |  |  |  |  |
| has-miR-500a-3p | 4,9,14 | 3 | 7.3 | 2.1 | 3.5 |  |  |  |  |  |  |  |  |  |  |  |
| has-miR-502-3p | 9,14,30 | 3 | 2.3 | 3.3 | 2.9 |  |  |  |  |  |  |  |  |  |  |  |
| has-miR-510 | 9,14,17 | 3 | 8.1 | 47.2 | 6.1 |  |  |  |  |  |  |  |  |  |  |  |
| has-miR-532-3p | 13,14,21 | 3 | 4.5 | 3.5 | 3.0 |  |  |  |  |  |  |  |  |  |  |  |
| has-miR-660-5p | 9,14,30 | 3 | 2.7 | 4.4 | 9.2 |  |  |  |  |  |  |  |  |  |  |  |
| miRNA expression fold-change upregulated in black and downregulated in red | | | | | | | | | | | | | | | | |

**Table S4**. Patients’ clinicopathological characters.

| Category | Value |
| --- | --- |
| No. patients | 45 |
| Age, median (range), yr | 58 (47-79) |
| BMI, mean ± SD, kg/m^2^ | 25.51 ± 2.78 |
| Male, No. (%) | 25 (55.56) |
| Tumor size, mean ± SD, cm | 4.98 ± 1.85 |
| Pathological stage, No. (%) |  |
| pT1a | 13 (28.89) |
| pT1b | 24 (53.33) |
| pT2 | 4 (8.89) |
| pT3 | 2 (4.44) |
| pT4 | 1 (2.22) |
| Fuhrman grade |  |
| G1 | 14 (31.11) |
| G2 | 28 (62.22) |
| G3 | 3 (6.67) |
| Local recurrence, No. (%) | 2 (4.44) |
| Metastasis, No. (%) | 1 (2.22) |
| BMI, body mass index; pT, pathological stage; G, grade. | |

**Table S5** miRNAs dysregulated in renal cell carcinoma and their involvement in cancer pathogenesis in other malignancies.

| miRNA | Cancer | Function | Target |
| --- | --- | --- | --- |
| **has-**miR-21-5p | Multiple cancers | Cellular growth, migration, invasion | PDCD4, RECK, TIMP3, MARKS, LRRF1P1, PTEN, SPROUTY2, TPM1, RHOB |
| **has-**miR-210-3p | Increased upon hypoxia | Increased cell survival and migration | Ephrin-A |
|  | Ovarian cancer | Regulate cell cycle | E2F3 |
|  | Multiple cancers | Response to hypoxia | MNT |
| **has-**miR-122-5p | HCC | Cell cycle and apoptosis | BCL-W, CCNG1, Cyclin G1 |
| **has-**miR-155-5p | Breast cancer | Tight junction dissolution, cell polarity, EMT | RHOA1 |
|  |  | Cell survival and chemoresistance | FOXO3 |
|  |  | Cell proliferation | SOCS1 |
|  | B-cell lymphoma | Cell proliferation | SHIP1 |
| **has-**miR-224-5p | HCC | Apoptotic cell death | API-5 |
| **has-**miR-138-5p | NSCLC | Cell proliferation | GPR124, PDK1 |
|  | HNSCC | Cellular death and invasion | RhoC |
|  | colorectal cancer | Metatstasis | TWIST2 |
| **has-**miR-204-5p | Gastric cancer | EMT | SIRT1, BCL2 |
| **has-**miR-218-5p | Gastric cancer | Cellular migration and invasion | ROBO1 receptor |
| **has-**miR-363-3p | HCC | Cell proliferation | S1PR1 |
|  | HNSCC | Cellular migration and invasion | podoplanin |
| **has-**miR-532-5p | Melanoma | Cell progression | RUNX3 |
| miR-200 family | Breast cancer | Repression during EMT sustains the process | ZEB1, ZEB2, TGFβ2 |
| HCC, hepatoma cell carcinoma; NSCLC, nonsmall-cell lung cancer; HNSCC, head and neck squamous cell carcinoma. hsa-miR-200 family includes hsa-miR-200a, hsa-miR-200b, hsa-miR-200c, hsa-miR-141, and hsa-miR-429. | | | |

**Table** **S6** Estimation of the hazard ratio on relative miRNAs expression and renal cell carcinoma cancer-specific survival (CSS).

| Reference | HR (95% CI) | *P* value |
| --- | --- | --- |
| **has-miR-210-3p** |  |  |
| Jung,2009 ^1^ | 0.882(0.366-2.12) | p = 0.779 |
| McCormick,2012 ^2^ | 3.01(1.39-6.51) | p = 0.005 |
| Neal,2010 ^3^ | 1.15(0.15-8.65) | p = 0.189 |
| Wotschofsky,2012 ^4^ | 0.39(0.12-1.23) | p = 0.109 |
| Current study | 6.85 (2.13-43.36) | p = 0.0045 |
| **has-miR-21-5p** |  |  |
| Faragalla,2012 ^5^ | 2.15(1.16-3.98) | p = 0.014 |
| Silva-Santos,2013 ^6^ | 2.47(1.30-4.71) | p = 0.006 |
| Vergho,2014 ^7^ | 1.31(1.12-1.53) | p = 0.0008 |
| Current study | 5.46 (2.02-53.39) | p = 0.0068 |
| **has-miR-141-3p** |  |  |
| Jung,2009 ^1^ | 1.08(0.45-2.59) | p = 0.864 |
| Silva-Santos,2013 ^6^ | 2.19(1.11-4.33) | p = 0.024 |
| Wotschofsky,2012 ^4^ | 1.26 (0.44-3.61) | p = 0.667 |
| Current study | 0.115 (0.004-0.183) | p = 0.0003 |
| **has-miR-200c-3p** |  |  |
| Jung,2009 ^1^ | 1.46(0.604-3.54) | p = 0.4 |
| Wotschofsky,2012 ^4^ | 0.56 (0.19-1.66) | p = 0.299 |
| Current study | 0.075 (0.013-0.428) | p = 0.0036 |
| **has-miR-429** |  |  |
| Jung,2009 ^1^ | 0.413(0.081-2.11) | p = 0.2669 |
| Current study | 0.183 (0.019-0.495) | p = 0.0068 |

Reference

1. Jung, M. *et al.* MicroRNA profiling of clear cell renal cell cancer identifies a robust signature to define renal malignancy. *J. Cell Mol. Med.* **13**,3918-3928 (2009).
2. McCormick, R.I. *et al.* miR-210 is a target of hypoxia-inducible factors 1 and 2 in renal cancer, regulates ISCU and correlates with good prognosis. *Br. J. Cancer.* **108**,1133-1142 (2012).
3. Neal, C.S. *et al.* The VHL-dependent regulation of microRNAs in renal cancer. *BMC Med.* **21**,64 (2010).
4. Wotschofsky, Z. *et al.* Identification of metastamirs as metastasis-associated microRNAs in clear cell renal cell carcinomas. *Int. J. Biol. Sci.* **8**, 1363-1374 (2012).
5. Faragalla, H. *et al.* The clinical utility of miR-21 as a diagnostic and prognostic marker for renal cell carcinoma. *J. Mol. Diagn.* **14**, 385-392 (2012).
6. Silva-Santos, R.M. *et al.* MicroRNA profile: a promising ancillary tool for accurate renal cell tumour diagnosis. *Br. J. Cancer* **109**, 2646-2653 (2013).
7. ergho, D.C. *et al.* Impact of miR-21, miR-126 and miR-221 as prognostic factors of clear cell renal cell carcinoma with tumor thrombus of the inferior vena cava. *PLoS One.* **9**,e109877 (2014).

**Table** **S7 Pooling data from eligible studies included for meta-analysis which reported prognosis of the five meta-signature miRNAs in RCC**

| Reference | miRNA | Method | Sample | No. of patients | Survival analysis | Follow-up |
| --- | --- | --- | --- | --- | --- | --- |
| Jung,2009 ^1^ | miR-210,miR-141,  miR-200c,miR-429 | qRT-PCR | Fresh-frozen tissues from nephrectomy | 84 | Univariate Cox  regression analysis | 26 months |
| McCormick,2012 ^2^ | miR-210 | qRT-PCR | Fresh-frozen tissues from nephrectomy | 30 | Kaplan Meier survival analysis | (1-100) months |
| Neal,2010 ^3^ | miR-210 | qRT-PCR | Hospital Tissue Bank facility | 31 | Kaplan Meier survival analysis | (1-140) months |
| Wotschofsky,2012 ^4^ | miR-210,miR-141,  miR-200c | qRT-PCR | Frozen tissue from nephrectomy | 87 | Univariate and multivariate  Cox regression analyses | (1-80) months |
| Faragalla,2012 ^5^ | miR-21 | qRT-PCR | formalin-fixed,  paraffin-embedded tissues | 107 | Univariate and multivariate  Cox regression analyses | 50 (1-192) months |
| Silva-Santos,2013 ^6^ | miR-21,miR-141 | qRT-PCR | Fresh-frozen tissues | 120 | Kaplan Meier survival analysis | 65 (1-120) months |
| Vergho,2014 ^7^ | miR-21 | qRT-PCR | paraffin embedded samples | 74 | Kaplan Meier survival analysis | (1-150) months |
